# Supplementary material for: A Likelihood Approach for Real-Time Calibration of Stochastic Compartmental Epidemic Models
Source: PLoS Comput Biol. 2017 Jan 17;13(1):e1005257. doi: 10.1371/journal.pcbi.1005257 (PMC5240920; doi:10.1371/journal.pcbi.1005257)
Supplement: S1 File — (TAR.GZ) [file pcbi.1005257.s014.tar.gz › HSPH_Online-SI-Revision/IPoi09/n100-severe/n100-severe_BENCH_table-8.pdf]

1	R0	{1.65278, 9.06793}	{1.26656, 3.29313}	{{1.26656, 3.29313}, {1.26656, 3.29313}}	{23.3679, 63.6838}	inf	990.	1078.89	{103., 1999.}	60.7921	0.354393	newly	963.	2637.79	{291., 4649.}	196.387	0.469664	Reff	1.60825	1.18561	{1.12263, 1.25647}	26.2796	ar	66342.	38775.1	{36839., 40290.}	41.5528
2	R0	{1.4321, 3.80644}	{2.15176, 6.48444}	{{1.4804, 3.12918}, {2.9194, 10.1535}}	{50.7018, 78.0351}	inf	86.	236.903	{43., 521.}	196.774	0.430512	newly	223.	531.516	{79., 1193.}	166.585	0.406608	Reff	1.42869	2.16828	{1.47546, 2.90823}	51.7666	ar	54217.	80700.7	{57082., 93226.}	48.8476
3	R0	{1.68388, 4.76028}	{1.90565, 4.7689}	{{1.31246, 2.23442}, {2.7566, 8.57964}}	{27.2336, 40.6373}	inf	40.	132.696	{11., 280.}	260.435	0.601605	newly	115.	350.717	{25., 819.}	239.565	0.627943	Reff	1.68211	2.08186	{1.31208, 2.8685}	32.6309	ar	68299.	69629.7	{104., 93169.}	32.4699
4	R0	{1.66014, 6.95383}	{2.18947, 7.31421}	{{1.56331, 3.72349}, {2.80387, 10.6536}}	{33.8535, 31.3479}	inf	267.	1642.42	{172., 3760.}	528.859	0.679266	newly	379.	2505.44	{331., 6006.}	570.601	0.718105	Reff	1.65028	2.20262	{1.56173, 2.84143}	35.0589	ar	66820.	82764.5	{65892., 93520.}	25.0752
5	R0	{1.69984, 11.2038}	{1.31535, 3.47086}	{{1.31535, 3.47086}, {1.31535, 3.47086}}	{22.6192, 69.0207}	inf	1644.	1785.89	{160., 3172.}	54.8797	0.307659	newly	1210.	3971.22	{421., 6239.}	243.985	0.523265	Reff	1.6312	1.17465	{0.990511, 1.30345}	27.9886	ar	68462.	44107.9	{42394., 45509.}	35.5732
6	R0	{1.65876, 6.66217}	{1.38393, 2.88882}	{{1.37363, 2.81014}, {1.37363, 2.81014}}	{16.5681, 56.6385}	inf	148.	1026.37	{175., 1958.}	601.902	0.775974	newly	251.	3348.59	{754., 6216.}	1241.3	1.04968	Reff	1.65251	1.34615	{1.26254, 1.45697}	18.5389	ar	67105.	50716.9	{48188., 59533.}	24.4215
7	R0	{1.69966, 5.89996}	{2.16116, 7.22018}	{{1.37363, 2.81014}, {2.80569, 11.317}}	{33.4123, 44.1296}	inf	95.	147.552	{14., 325.}	107.84	0.40545	newly	161.	265.086	{23., 755.}	114.575	0.402904	Reff	1.69633	2.25775	{1.52858, 2.93659}	37.7678	ar	69396.	81231.6	{60881., 93631.}	23.6947
8	R0	{1.53428, 6.3734}	{1.24751, 2.5364}	{{1.24751, 2.5364}, {1.24751, 2.5364}}	{18.6909, 60.2033}	inf	389.	841.333	{196., 1652.}	131.054	0.333217	newly	553.	2873.27	{747., 5454.}	419.578	0.640894	Reff	1.51552	1.18491	{1.10274, 1.23092}	21.815	ar	59855.	36978.9	{35665., 38611.}	38.2193
9	R0	{1.58939, 3.48608}	{1.48146, 4.65633}	{{1.06836, 1.81005}, {2.48103, 11.1331}}	{28.1231, 69.2304}	inf	10.	20.0833	{0., 61.}	151.389	0.578566	newly	29.	44.0833	{0., 182.}	137.835	0.768401	Reff	1.58888	1.75876	{1.0674, 2.61166}	29.9916	ar	62575.	52064.1	{22., 90582.}	46.6178
10	R0	{1.58528, 5.5593}	{1.25656, 3.82585}	{{1.25656, 3.82585}, {1.25656, 3.82585}}	{20.7358, 31.1811}	inf	179.	373.667	{27., 926.}	143.886	0.425888	newly	327.	833.056	{66., 2055.}	181.668	0.441265	Reff	1.57528	1.23216	{1.19236, 1.25425}	21.7813	ar	63039.	37868.4	{36109., 39107.}	39.9286
11	R0	{1.58305, 4.62721}	{1.27657, 1.95709}	{{1.27121, 1.91565}, {1.31246, 2.23442}}	{19.3599, 57.7047}	inf	50.	206.071	{30., 543.}	329.286	0.60753	newly	133.	1204.61	{251., 3132.}	815.548	0.91814	Reff	1.57961	1.26509	{1.24026, 1.295}	19.9116	ar	63222.	38711.1	{38183., 43488.}	38.7696
12	R0	{1.70234, 9.34626}	{1.29588, 3.74196}	{{1.25474, 3.26445}, {1.35758, 4.45823}}	{23.8768, 59.963}	inf	872.	1181.71	{221., 2043.}	71.1746	0.339217	newly	836.	2539.57	{590., 4555.}	220.164	0.509584	Reff	1.66487	1.2284	{1.10988, 1.33184}	26.2166	ar	68213.	42596.7	{36441., 48550.}	37.5534
13	R0	{1.68556, 9.74954}	{1.31535, 3.47086}	{{1.31535, 3.47086}, {1.31535, 3.47086}}	{21.9636, 64.3998}	inf	1232.	1785.89	{160., 3172.}	81.2771	0.338644	newly	1100.	3971.22	{421., 6239.}	276.162	0.555459	Reff	1.63051	1.17465	{0.990511, 1.30345}	27.9581	ar	68426.	44107.9	{42394., 45509.}	35.5393
14	R0	{1.59876, 9.78897}	{1.26656, 3.29313}	{{1.26656, 3.29313}, {1.26656, 3.29313}}	{20.7786, 66.3588}	inf	1006.	1400.84	{174., 2362.}	76.5408	0.325482	newly	885.	3285.95	{530., 5220.}	282.676	0.542204	Reff	1.55367	1.14812	{1.05846, 1.25103}	26.1027	ar	63553.	38775.1	{36839., 40290.}	38.9878
15	R0	{1.43577, 5.12819}	{1.25442, 3.26804}	{{1.25474, 3.26445}, {1.25474, 3.26445}}	{12.6311, 36.273}	inf	217.	722.857	{159., 1477.}	250.143	0.503596	newly	384.	1868.33	{452., 3606.}	395.449	0.628158	Reff	1.42469	1.20258	{1.1407, 1.2387}	15.59	ar	53259.	37452.7	{36234., 38444.}	29.6782
16	R0	{1.58866, 8.73191}	{1.41875, 4.96977}	{{1.41571, 4.94504}, {1.41571, 4.94504}}	{10.6952, 43.085}	inf	604.	1404.52	{338., 2665.}	157.213	0.41967	newly	587.	2420.13	{683., 4462.}	324.909	0.581499	Reff	1.56477	1.34272	{1.27437, 1.40053}	14.1905	ar	64349.	52492.5	{51631., 53185.}	18.4253
17	R0	{1.43012, 3.8163}	{1.30871, 2.14092}	{{1.27121, 1.91565}, {1.4804, 3.12918}}	{10.1438, 45.0417}	inf	64.	234.69	{30., 544.}	288.362	0.55744	newly	190.	1275.14	{84., 3132.}	585.209	0.747847	Reff	1.42695	1.30135	{1.24026, 1.47168}	9.79332	ar	53353.	43228.6	{38435., 56911.}	21.1592
18	R0	{1.47965, 5.16472}	{1.45744, 3.55954}	{{1.43579, 3.38828}, {1.47608, 3.83841}}	{3.53223, 32.3547}	inf	233.	677.25	{46., 1666.}	210.789	0.556795	newly	443.	2012.58	{163., 4708.}	372.492	0.719297	Reff	1.47015	1.42646	{1.35392, 1.4544}	5.01954	ar	56086.	53664.5	{53040., 56863.}	7.73351
19	R0	{1.57191, 5.2314}	{2.14894, 8.52985}	{{1.47608, 3.83841}, {2.68687, 12.2271}}	{38.5291, 70.5929}	inf	225.	539.911	{83., 1202.}	168.452	0.4007	newly	437.	784.232	{127., 1837.}	119.577	0.348905	Reff	1.563	2.14124	{1.47193, 2.68426}	38.8477	ar	63119.	80199.7	{57474., 91336.}	29.5246
20	R0	{1.54435, 5.32911}	{2.47367, 14.4591}	{{1.93539, 9.63533}, {2.93183, 18.8445}}	{60.1756, 171.322}	inf	191.	548.065	{81., 1083.}	212.194	0.464885	newly	372.	381.806	{53., 708.}	67.1609	0.37004	Reff	1.53601	2.48213	{2.16185, 2.90386}	61.5958	ar	61870.	88264.7	{83786., 93510.}	42.6615
21	R0	{1.57932, 4.90031}	{1.96281, 5.98371}	{{1.56331, 3.72349}, {2.65613, 9.78983}}	{26.8121, 43.8813}	inf	57.	527.	{110., 1003.}	829.96	0.876675	newly	102.	1083.69	{185., 2357.}	967.044	0.918122	Reff	1.57657	2.07619	{1.54849, 2.77201}	33.399	ar	63595.	76827.	{61920., 92516.}	27.1157
22	R0	{1.47044, 4.07694}	{1.27747, 2.74826}	{{1.19264, 2.01465}, {1.42949, 4.01966}}	{14.3302, 36.3478}	inf	83.	558.231	{14., 1254.}	602.688	0.810827	newly	207.	1841.19	{28., 4315.}	819.008	0.921573	Reff	1.46579	1.25647	{1.13215, 1.42722}	15.1436	ar	55895.	38894.7	{28697., 53995.}	32.1819
23	R0	{1.64997, 3.34546}	{2.05829, 4.81632}	{{1.31246, 2.23442}, {2.72208, 6.87793}}	{32.6631, 57.7591}	inf	9.	30.0147	{0., 75.}	285.784	0.737864	newly	51.	100.765	{0., 292.}	165.859	0.814485	Reff	1.64943	2.16436	{1.43694, 2.86874}	36.4644	ar	66404.	65252.	{5., 92962.}	42.7489
24	R0	{1.67405, 4.50254}	{2.15114, 5.08361}	{{1.27121, 2.14605}, {2.85474, 9.2843}}	{39.1878, 46.1061}	inf	14.	6.67	{0., 21.}	82.5	1.16118	newly	40.	42.04	{0., 140.}	136.75	1.44543	Reff	1.6737	2.15088	{1.27117, 2.8524}	39.1922	ar	68017.	43842.5	{1., 92520.}	62.1714
25	R0	{1.50663, 3.1286}	{2.03394, 9.87388}	{{1.22544, 4.06746}, {2.7566, 17.2462}}	{42.3996, 216.924}	inf	3.	3.01	{0., 8.}	106.333	0.740359	newly	20.	7.87	{0., 17.}	103.65	1.53557	Reff	1.50652	2.03383	{1.22543, 2.75613}	42.4002	ar	58823.	37457.2	{1., 91225.}	73.5205
26	R0	{1.69822, 6.34992}	{2.30913, 13.3454}	{{1.68297, 7.5059}, {2.88523, 18.8445}}	{38.3871, 113.361}	inf	141.	213.77	{28., 556.}	107.371	0.398428	newly	238.	187.426	{15., 401.}	71.8487	0.508148	Reff	1.69328	2.33188	{1.82808, 2.86581}	40.287	ar	69816.	83850.	{75012., 93140.}	24.0529
27	R0	{1.55869, 4.92988}	{2.42123, 12.7637}	{{1.739, 7.10524}, {2.8939, 16.8013}}	{55.3372, 158.904}	inf	86.	194.095	{45., 394.}	152.344	0.39507	newly	214.	167.492	{38., 357.}	61.4597	0.406532	Reff	1.55537	2.42478	{1.75319, 2.90675}	55.8973	ar	62344.	86507.	{71225., 93369.}	38.7575
28	R0	{1.46085, 6.06899}	{1.43579, 3.38828}	{{1.43579, 3.38828}, {1.43579, 3.38828}}	{1.71544, 44.1706}	inf	547.	1576.91	{308., 2789.}	207.567	0.504192	newly	795.	4430.52	{938., 7329.}	467.471	0.71458	Reff	1.43891	1.35259	{1.28317, 1.41662}	5.99901	ar	55484.	53839.5	{53200., 54612.}	2.96388
29	R0	{1.63017, 6.25164}	{1.37607, 3.33026}	{{1.36035, 3.22945}, {1.47488, 3.83841}}	{15.7386, 46.7298}	inf	248.	576.	{105., 1279.}	162.174	0.387811	newly	360.	1708.52	{351., 3916.}	381.706	0.558169	Reff	1.6206	1.34735	{1.30595, 1.37357}	16.8613	ar	66231.	49095.7	{47127., 56390.}	25.872
30	R0	{1.45173, 5.68165}	{2.03927, 11.8121}	{{1.85376, 9.90957}, {2.42074, 15.7308}}	{40.5412, 108.152}	inf	352.	964.339	{132., 2137.}	202.773	0.467671	newly	541.	736.898	{113., 1557.}	87.2772	0.375977	Reff	1.43681	2.07182	{1.80126, 2.41043}	44.1965	ar	55280.	81342.1	{75206., 89582.}	47.1456
31	R0	{1.55808, 2.77316}	{2.0357, 10.7496}	{{1.17434, 5.03398}, {2.7943, 18.2127}}	{40.9606, 287.749}	inf	4.	3.3	{0., 9.}	99.5	0.768033	newly	23.	6.85	{0., 13.}	95.6957	1.52405	Reff	1.55797	2.03559	{1.1743, 2.7941}	40.9607	ar	60940.	40929.2	{1., 92228.}	71.8021
32	R0	{1.56797, 3.90327}	{2.42607, 9.04301}	{{1.61691, 4.41442}, {2.93597, 11.6897}}	{56.2172, 132.941}	inf	24.	41.697	{6., 93.}	114.268	0.421791	newly	62.	62.5303	{5., 118.}	70.9433	0.454304	Reff	1.56717	2.49752	{1.8474, 2.93497}	60.3029	ar	62228.	84186.7	{74024., 93702.}	42.9929
33	R0	{1.70027, 5.43261}	{2.31206, 6.23915}	{{1.88643, 4.56746}, {2.9751, 8.62388}}	{36.2491, 25.5595}	inf	66.	146.356	{33., 374.}	146.148	0.418602	newly	163.	369.288	{74., 918.}	154.32	0.439931	Reff	1.69787	2.40489	{1.88469, 2.97067}	41.8701	ar	68798.	84857.2	{76085., 93916.}	26.9411
34	R0	{1.61668, 6.86192}	{2.02692, 13.251}	{{1.40191, 5.7404}, {2.36153, 17.2664}}	{28.6909, 97.4773}	inf	325.	776.05	{193., 1579.}	162.918	0.374755	newly	465.	542.517	{132., 1344.}	77.9606	0.360583	Reff	1.60218	2.06113	{1.68749, 2.35688}	30.9714	ar	64693.	80302.9	{71623., 87518.}	27.5801
35	R0	{1.55955, 3.45071}	{2.10999, 9.20722}	{{1.31535, 3.47086}, {2.85273, 14.4607}}	{41.8107, 171.059}	inf	11.	11.7903	{2., 23.}	80.4985	0.427992	newly	53.	19.9032	{1., 52.}	80.8278	0.978048	Reff	1.55925	2.25106	{1.54358, 2.91756}	47.8145	ar	61783.	73212.2	{191., 93684.}	43.6075
36	R0	{1.65068, 4.91307}	{2.33599, 11.4969}	{{1.75445, 7.55154}, {2.85582, 15.2798}}	{43.4142, 135.493}	inf	26.	46.5645	{5., 90.}	113.834	0.370342	newly	80.	40.9032	{5., 82.}	57.2177	0.500474	Reff	1.64972	2.45279	{2.00199, 2.9086}	49.1893	ar	66453.	87159.6	{79712., 93459.}	31.9372
37	R0	{1.46131, 3.06714}	{1.31755, 1.38915}	{{1.30222, 1.35944}, {1.36566, 1.47098}}	{9.83756, 54.7086}	inf	18.	68.3333	{0., 179.}	336.831	0.908588	newly	72.	793.963	{0., 1827.}	1041.1	1.26849	Reff	1.45985	1.317	{1.28672, 1.36425}	9.78541	ar	56452.	36131.	{16., 48319.}	35.997
38	R0	{1.71034, 5.2546}	{2.33541, 8.85112}	{{1.74592, 5.58477}, {2.8186, 12.0293}}	{39.0844, 73.7684}	inf	60.	121.873	{13., 280.}	140.582	0.435076	newly	144.	185.81	{23., 472.}	92.6587	0.431764	Reff	1.70807	2.37532	{1.80256, 2.92202}	41.585	ar	69816.	83432.4	{70681., 93536.}	26.5397
39	R0	{1.43565, 5.57989}	{1.47261, 3.82014}	{{1.47608, 3.83841}, {1.47608, 3.83841}}	{2.88901, 31.5373}	inf	400.	2736.35	{499., 4970.}	585.76	0.747147	newly	680.	6306.46	{1467., 10137.}	827.421	0.892959	Reff	1.4178	1.32434	{1.16857, 1.45155}	7.38385	ar	54407.	56490.9	{55996., 57609.}	4.70952
40	R0	{1.40327, 4.00764}	{1.47231, 2.7368}	{{1.43965, 2.60601}, {1.43965, 2.60601}}	{4.92016, 33.3685}	inf	122.	508.821	{57., 1024.}	338.612	0.622267	newly	269.	2275.64	{258., 4450.}	755.789	0.837071	Reff	1.39731	1.46037	{1.4012, 1.5719}	4.59719	ar	51976.	56486.1	{53240., 63888.}	8.67736
41	R0	{1.63612, 4.26741}	{2.45862, 4.71149}	{{1.89552, 3.26011}, {2.86957, 5.76688}}	{50.8607, 20.7654}	inf	38.	117.018	{4., 226.}	236.934	0.648827	newly	108.	617.105	{36., 1156.}	493.713	0.87293	Reff	1.6346	2.53392	{2.03637, 2.86306}	55.0176	ar	65029.	81150.5	{75222., 93071.}	42.3334
42	R0	{1.57426, 6.50271}	{1.52539, 5.96853}	{{1.41571, 4.94504}, {1.85533, 9.28761}}	{9.51699, 23.1048}	inf	287.	784.475	{62., 1724.}	204.155	0.475793	newly	463.	1188.68	{107., 2795.}	187.478	0.454529	Reff	1.56141	1.54332	{1.38667, 1.85292}	10.2184	ar	62522.	61699.5	{53536., 75257.}	12.6448
43	R0	{1.37818, 5.37636}	{1.32768, 3.8923}	{{1.26656, 3.29313}, {1.67405, 7.28757}}	{10.1044, 38.268}	inf	406.	1073.54	{96., 2081.}	199.64	0.482191	newly	728.	2031.77	{215., 3686.}	205.928	0.478243	Reff	1.35702	1.33203	{1.17651, 1.64037}	12.6613	ar	50098.	47766.	{37257., 68248.}	26.7635
44	R0	{1.66606, 4.92641}	{2.1307, 7.30139}	{{1.37363, 3.22945}, {2.80569, 11.0005}}	{35.4893, 63.8392}	inf	32.	49.1961	{9., 100.}	105.208	0.406837	newly	79.	78.6667	{14., 148.}	77.2648	0.450454	Reff	1.66501	2.35491	{1.61594, 2.91879}	44.1373	ar	67066.	79749.3	{56595., 93548.}	33.6394
45	R0	{1.623, 5.9449}	{1.37847, 2.84659}	{{1.37363, 2.81014}, {1.37363, 2.81014}}	{15.0665, 52.1171}	inf	133.	436.913	{51., 924.}	258.058	0.518102	newly	254.	1643.3	{253., 3348.}	556.248	0.688135	Reff	1.61652	1.35822	{1.32274, 1.37033}	15.979	ar	64554.	49469.3	{48188., 49782.}	23.3676
46	R0	{1.65235, 5.51234}	{1.44087, 4.96916}	{{1.22544, 3.07157}, {1.82266, 8.17412}}	{24.4216, 49.5383}	inf	70.	145.576	{35., 343.}	143.377	0.379271	newly	154.	313.242	{40., 742.}	150.748	0.431842	Reff	1.64944	1.56723	{1.21636, 2.58626}	26.6648	ar	66509.	51867.5	{34542., 90764.}	35.4667
47	R0	{1.62138, 8.82208}	{1.30094, 3.11158}	{{1.30094, 3.11158}, {1.30094, 3.11158}}	{19.7634, 64.7296}	inf	1152.	1390.82	{119., 2953.}	70.5984	0.349751	newly	1173.	3588.47	{326., 7065.}	225.009	0.515546	Reff	1.57308	1.20035	{1.06993, 1.29352}	23.6944	ar	65598.	42208.9	{41355., 43623.}	35.6551
48	R0	{1.64073, 6.69261}	{1.61344, 4.02363}	{{1.56331, 3.72349}, {1.64953, 4.30394}}	{6.35847, 41.3623}	inf	217.	414.697	{33., 829.}	134.674	0.420601	newly	314.	1223.27	{92., 2780.}	319.031	0.566873	Reff	1.63171	1.65048	{1.52981, 1.96178}	9.38515	ar	66438.	65947.2	{61859., 78539.}	8.6894
49	R0	{1.65556, 3.796}	{2.13986, 6.67946}	{{1.30094, 2.60601}, {2.85474, 13.1646}}	{38.534, 87.4435}	inf	5.	5.47	{0., 15.}	115.	0.883617	newly	9.	26.35	{0., 70.}	305.222	1.22406	Reff	1.65538	2.13966	{1.30091, 2.85226}	38.5341	ar	66575.	43946.	{1., 92455.}	63.3894
50	R0	{1.56828, 3.65713}	{2.20426, 4.34144}	{{1.48799, 2.26472}, {2.82901, 6.03877}}	{42.758, 35.4448}	inf	37.	74.4769	{0., 146.}	156.59	0.709111	newly	103.	356.477	{0., 751.}	287.199	0.858353	Reff	1.56703	2.23869	{1.48737, 2.82672}	44.9882	ar	62761.	71250.8	{17., 92720.}	43.7729
